# Plant and soil microbial composition legacies following indaziflam herbicide treatment

**DOI:** 10.3389/fmicb.2024.1450633

**Published:** 2024-12-18

**Authors:** Ember Sienna Bradbury, Hannah Holland-Moritz, Amy Gill, Caroline A. Havrilla

**Affiliations:** ^1^Department of Forestry and Rangeland Stewardship, Colorado State University, Fort Collins, CO, United States; ^2^Department of Natural Resources and the Environment, University of New Hampshire, Durham, NC, United States

**Keywords:** indaziflam, cheatgrass, restoration ecology, soil microbial community, Intermountain West (US)

## Abstract

Land stewards in dryland ecosystems across the western U.S. face challenges to manage the exotic grass *Bromus tectorum* (cheatgrass), which is a poor forage, is difficult to remove, and increases risk of catastrophic fire. Managers may consider using indaziflam (Rejuvra™), a relatively new pre-emergent herbicide, which may reduce cheatgrass cover within drylands. However, few studies have explored the effects of indaziflam on non-target organisms. We tested how indaziflam application impacted cover and biomass of native and exotics within the plant community and composition and diversity of the soil microbiome by comparing untreated and treated arid shrubland sites in Boulder County, Colorado, USA. We found that indaziflam application decreased cheatgrass cover by as much as 80% and increased native plant cover by the same amount. Indaziflam application also was associated with increased soil nitrate (NO_3_^−^), decreased soil organic matter, and had a significant effect on the composition of the soil microbiome. Microbial community composition was significantly related to soil NO_3_^−^, soil organic matter, soil pH, and native species and cheatgrass biomass. An indicator species analysis suggested that indaziflam application shifted microbial communities. In untreated sites, ammonia-oxidizing bacteria *Nitrosomonadaceae* and nitrogen-digesting *Opitutaceae* and the fungi *Articulospora proliferata* were found. While in treated sites, ammonia-oxidizing archaea which are associated with intact drylands, *Nitrososphaeraceae* and toxin digesters and acidic-soil species *Sphingomonas* and *Acidimicrobiia* were significantly associated. Overall, these results demonstrate that indaziflam application can increase native plant recruitment, while also affecting soil properties and the soil microbiome. The findings from this study can be used to inform decision-making during dryland restoration planning process as indaziflam use may have benefits and unknown long-term consequences for the biogeochemistry and microbial ecology of the system.

## Introduction

Cheatgrass now occupies over 54 million acres in the Intermountain West (the region of the United States between the Rocky Mountains, Cascades, and Sierra Nevada’s but not including the Pacific Coast) after its introduction as a contaminant in grain during the 1900s (Pierson and Mack, 1990; Sebastian et al., 2017). Cheatgrass is an early seeding grass which outcompetes native plant species, resulting in loss of diversity, disruptions of historic grazing regimes, and increased risk of catastrophic wildfire (Balch et al., 2013; Pilliod et al., 2017). These impacts have driven land managers to seek strategies for reducing cheatgrass populations, but targeted grazing, prescribed burning, and use of other herbicides have all had limited success (Lehnhoff et al., 2019; Mack, 2010; Perryman et al., 2020).

Indaziflam (marketed as Rejuvra®/Esplanade® by Bayer CropScience) is a relatively new herbicide that was approved in 2010 for use to control invasive annual grasses on rangeland and open space. Indaziflam is a fluoroalkyl triazine-containing herbicide that inhibits cellulose synthesis (Brabham et al., 2014). Indaziflam application has shown reduction of cheatgrass (Clark et al., 2023; Meyer-Morey et al., 2021; Sebastian et al., 2017). However, weed control with indaziflam may also impact non-target taxa (Meyer-Morey et al., 2021; Sebastian et al., 2020; Strilbytska et al., 2022). Thus far, treatment with indaziflam has shown positive (Clark et al., 2023; Seshadri et al., 2018) and negative (Clenet et al., 2019; Fowers and Mealor, 2020; Meyer-Morey et al., 2021) effects on native plant cover and diversity. While Seshadri et al. (2018) found that indaziflam increased native forb diversity and floral resources for pollinators, Meyer-Morey et al. (2021) saw reduction in cover of target invasive annual mustards (Alyssum spp.) and also decreased native forb diversity 2 years following treatment in a sagebrush steppe ecosystem. Given these limited and conflicting results, additional study about indaziflam effects on native plant communities and non-target organisms following treatment is needed.

Herbicides may also impact soil microbial communities and nutrient cycling (Asad et al., 2017; Caggìa et al., 2023; Sebastian et al., 2020; Van Bruggen et al., 2021), although few studies have investigated indaziflam’s effect on these processes. Soil microbes mediate key ecosystem functions (Fierer et al., 2021; Neilson et al., 2017; Wang and Li, 2019), and herbicide treatments can have variable effects on them (e.g., Van Bruggen et al., 2021). For example, the broad-spectrum systemic herbicide glyphosate interferes with nitrogen metabolism in some soil bacteria, while others can digest it (Helander et al., 2012; Huang et al., 2017; Ruuskanen et al., 2023). In a study that examined indaziflam impacts on soil properties, application shifted soil carbon and nitrogen mineralization rates 11 days after treatment (Koçak et al., 2021). However, to our knowledge, there have been no studies of how indaziflam impacts soil microbial community composition in natural grassland systems.

The central goal of this study was to explore the effects of indaziflam on soil microbial and native plant communities to improve management planning and practice. The major research objectives of this project were to:

1. Observationally evaluate the effects of indaziflam on non-target soil microorganisms and native plants by comparing community composition in areas that have been treated with indaziflam herbicide with untreated controls.
2. Assess whether indaziflam effects on soil microbes and native plants vary across

- Ecological gradients (e.g., soil texture, ecological site type, and pre-treatment exotic grass cover)
- Time since application.

3. Explore relationships among exotic plant, native plant, and soil community composition in treatment versus control plots to infer potential mechanisms whereby indaziflam could affect the soil microbial communities.

Incomplete understanding of the effects of indaziflam on non-target organisms in natural systems currently limits the capacity of land stewards to weigh the potential benefits and risks of indaziflam. The findings from this study can support land manager decision-making by increasing understanding of indaziflam effects on native plant and soil microbial communities relative to controls.

## Methods

### Site description

We collected soil samples and data describing soil physical characteristics and plant communities from seven sites within arid foothill shrubland 7,000 ft. above sea level in Boulder County Parks and Open Space (BCPOS) in Boulder, Colorado USA in June 2022 (Caudle et al., 2013; Soil Survey Staff, 2024). Soil and vegetation data were collected at seven sites spanning a 5-year gradient of time since indaziflam treatment (2017–2022). Each site contained two paired plots [50×50-m plots one treated with indaziflam (T) and one untreated (U)] for a total of 16 plots. Paired plots were selected to have similar potential vegetation (ESD), soil texture, and slope (Table 1) to each plot. All sites had an average annual temperature of 7.9°C and an average annual precipitation of 516 mm (PRISM Climate Group, Oregon State University, 2014).

**Table 1:** Site characteristics and herbicide application information for each site.

Site ID	Site name	Year treated	Average Soil pH	Soil texture
RABB22	Rabbit Mountain	2022	6.6	Very stony sandy loam and clay loam
DORO21	Dorothy Ellen	2021	7.08	Very stony sandy loam
DORO20	Dorothy Ellen	2020	7.13	Very stony sandy loam
TREVA22	Trevarton	2022	7.16	Gravelly sandy loam
TREVA19	Trevarton	2019	6.35	Gravelly sandy loam
TREVA18	Trevarton	2018	5.75	Gravelly sandy loam
TREVA17	Trevarton	2017	6.46	Gravelly sandy loam

Soil texture information from USDA Web Soil Survey tool (Soil Survey Staff, 2024).

### Plant community monitoring

**Figure 1:**
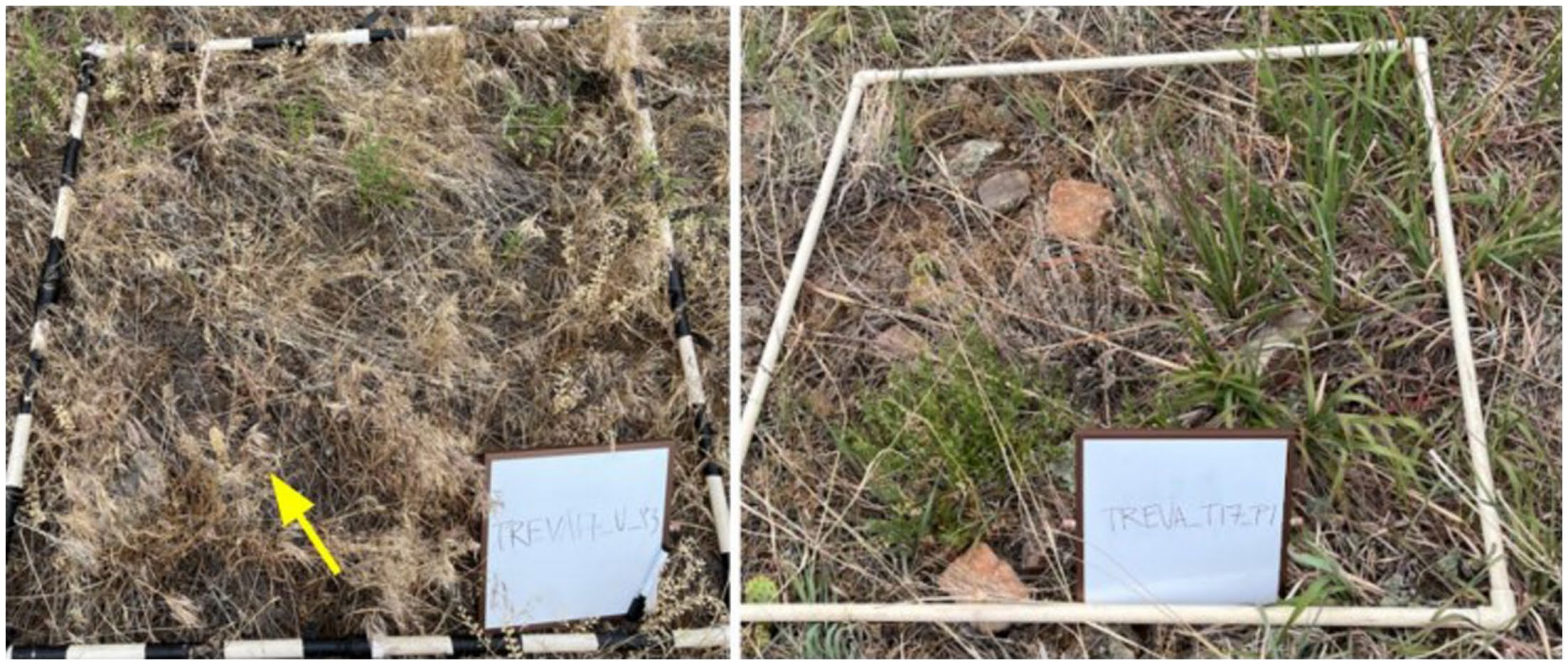
Example photographs of untreated (left) and treated (right) plots taken within the Trevarton site. Untreated plots were generally noticeably covered in cheatgrass (yellow arrow pointing to an example), while treated plots usually had fallen cheatgrass, but mostly native grasses and forbs growing within the plot.

To explore how plant communities responded to herbicide treatment, we collected plant cover and biomass data for native and exotic plant communities from indaziflam-treated and untreated plots. Vegetation data were collected from three, 1×1-m subplots within each plot using a quadrat (Figure 1, Supplementary Figure S1). Subplots were first photographed, and a general description of the plant community and site type was recorded. Within each subplot, we collected plant data on (1) species-level native plant cover (% cover) using a cover estimator and visual estimate and biomass (ounces) for grasses, forbs, shrubs, and bare ground, (2) exotic plant cover and biomass, (3) native plant diversity (e.g., species richness and Shannon diversity) through identification of each species present by a trained botanist and using the USDA, NCRS (2022) database citation,^1^ and (4) ground level thatch biomass, depth, and cover for native plants and cheatgrass (mm measured at thickest point with a ruler). Data were collected from all plots in June 2022. Some of the sites shared the same control plots, so vegetation monitoring was conducted only once for the shared sites.

### Soil physical sampling and analyses

Three soil samples (500 g) were collected to a depth of 10 cm from each subplot within each plot using a sterilized trowel (Supplementary Figure S1). Soil samples collected from each subplot (*n* = 3 each) were then pooled and homogenized. All samples were air-dried and stored in paper bags at room temperature prior to analysis. Samples were then sent to the Colorado State University Soil and Plant Testing Lab^2^ where organic matter content, electrical conductivity, pH, and nitrate (NO_3_^−^) were measured. Organic matter was measured using the loss on ignition method (Mehlich, 1984; Ball, 1964). Bulk density was measured with a mass per volume calculation using intact oven-dried 200 mL subsampled cores (Håkansson, 1990; Håkansson and Lipiec, 2000). Electrical conductivity and pH were measured using a 1:1 soil to water suspension (Sparks, 1996). Finally, nitrate was measured using a 2 M KCl extraction (Matsumura and Witjaksono, 1999).

### Soil microbial sampling

Soil samples for microbial analysis were collected from the same subplots (Supplementary Figure S1). For these samples, 10 g of soil was collected from three points within each subplot to a depth of 10 cm using a metal trowel that was rinsed in ethanol between plot sampling events (Penton et al., 2016). All samples were collected in sterile Whirl-Paks™ after sifting roots and debris out and homogenized on site, placed on dry ice during transport, and then stored in a − 40°C freezer until further analysis. Before conducting microbial analysis, soils were sieved through a 3.35 mm sieve.

### Soil DNA extraction, PCR, and gene amplicon sequencing

See Appendix 1 for a full microbial analysis methodology. First, DNA was extracted from 0.25 g of soil using the Qiagen DNeasy PowerSoil Pro Kit (Qiagen, Hilden, Germany) following the manufacturer’s instructions. Blank samples were used as negative controls. Next, we used primers (515F/806R 16S and ITS1-F/ITS2 ITS) to amplify the V4 region of the 16S rRNA gene for bacteria and archaea, and the ITS gene region for fungi (Bellemain et al., 2010; Iwen et al., 2002; Parada et al., 2016; Walters et al., 2016). Each sample was assigned a 12-bp barcode, homogenized, and then randomly assigned a location on a 96-well plate. The reactions were run in duplicate and then combined. SequalPrep Normalization Kit was used to normalize. DNA extraction was done in the Dryland Ecology and Management Lab at Colorado State University, while PCR and sequencing were done at the CIRES Microbial Community Sequencing Laboratory at the University of Colorado Boulder. Samples were then sequenced with the Illumina MiSeq platform. For 16S, a 300-cycle kit and, for ITS, a 500-cycle kit were used, paired-end (PE) for both. After sequencing, idemp (idemp^3^) was used to demultiplex the samples according to their specific barcodes. We used cutadapt (Martin, 2011) for cleaning. We then used the dada2 package in R (Callahan et al., 2016) to characterize the microbial communities in each sample. After using the filertaAndTrim() function (settings: 16S truncLen = c(150,150), ITS truncLen = c(200,220), maxEE = (2,2), truncQ = 2, rm.phix = T) (see Supplementary Figure S3 for quality read plots), learning error rates, and merging pairs, we used a Bayesian taxonomic identifier (Wang et al., 2007) as implemented in the dada2 package to assign a taxonomy based on UNITE (Oct. 2021 release for ITS) and Silva (v 138.1 for 16S).

### Statistical analyses

Soil microbiome data were filtered and rarified prior to statistical analysis. Amplicon sequence variants (ASVs) that were highly abundant in negative control samples were filtered out after verifying that these ASVs were not highly abundant in the sample data.

All statistical analyses were performed in R version 4.2.2 (R Core Team, 2023). We used generalized linear mixed effects models (GLMMs) with the lme4 package (Bates et al., 2014). Separate models were built for the following response variables: cheatgrass cover (%), cheatgrass biomass (ounces per m^2^), cheatgrass thatch depth (cm), total native herbaceous plant cover (%), total native forb cover (%), total native shrub cover (%), native species richness, and soil mineral nutrient levels of interest (i.e., organic matter, NO_3_^−^ (ppm), pH, and others). In each model, indaziflam treatment (i.e., treated vs. control) was included as fixed effects, and Site_ID was included as a random effect. The significance of individual terms (*p* < 0.05) included in final models was estimated using a Wald Type II Χ2 test (‘ANOVA’ function, car package; Fox and Weisberg, 2011). For significant variables, we used planned contrasts to explore differences in group means among levels using the ‘emmeans’ function (package emmeans; Lenth, 2017).

Permutational analysis of variance (PERMANOVA) tests were then conducted using the vegan, pairwiseAdonis, and smartsnp packages (Arbizu, 2020; Herrando‐Pérez et al., 2021; Oksanen, 2020; Polanco-Martínez et al., 2020). In these models, treatment (treated with indaziflam or not treated) was the predictor variable, and Bray–Curtis dissimilarity matrices of vegetation and soil variables were the response variables. PERMANOVAs were nested by site. We first calculated the Shannon index and species richness using the diversity() and specnumber() functions of the vegan package (Oksanen, 2020).

We then used ANOVA (analysis of variance) tests to compare the Shannon indices and species richness (Fox and Weisberg, 2011). To parse apart if soil and ecological variables had a related effect on microbial diversity and community composition, we also conducted a multiple regression on distance matrices (MDRM) test (Goslee and Urban, 2007). Finally, we performed an indicator species analysis to determine taxa indicative of treated and untreated conditions. For this analysis, we used the multipatt() function of the indicspecies package (De Cáceres et al., 2010).

## Results

### Cheatgrass versus native plant species responses to indaziflam treatment

**Figure 2:**
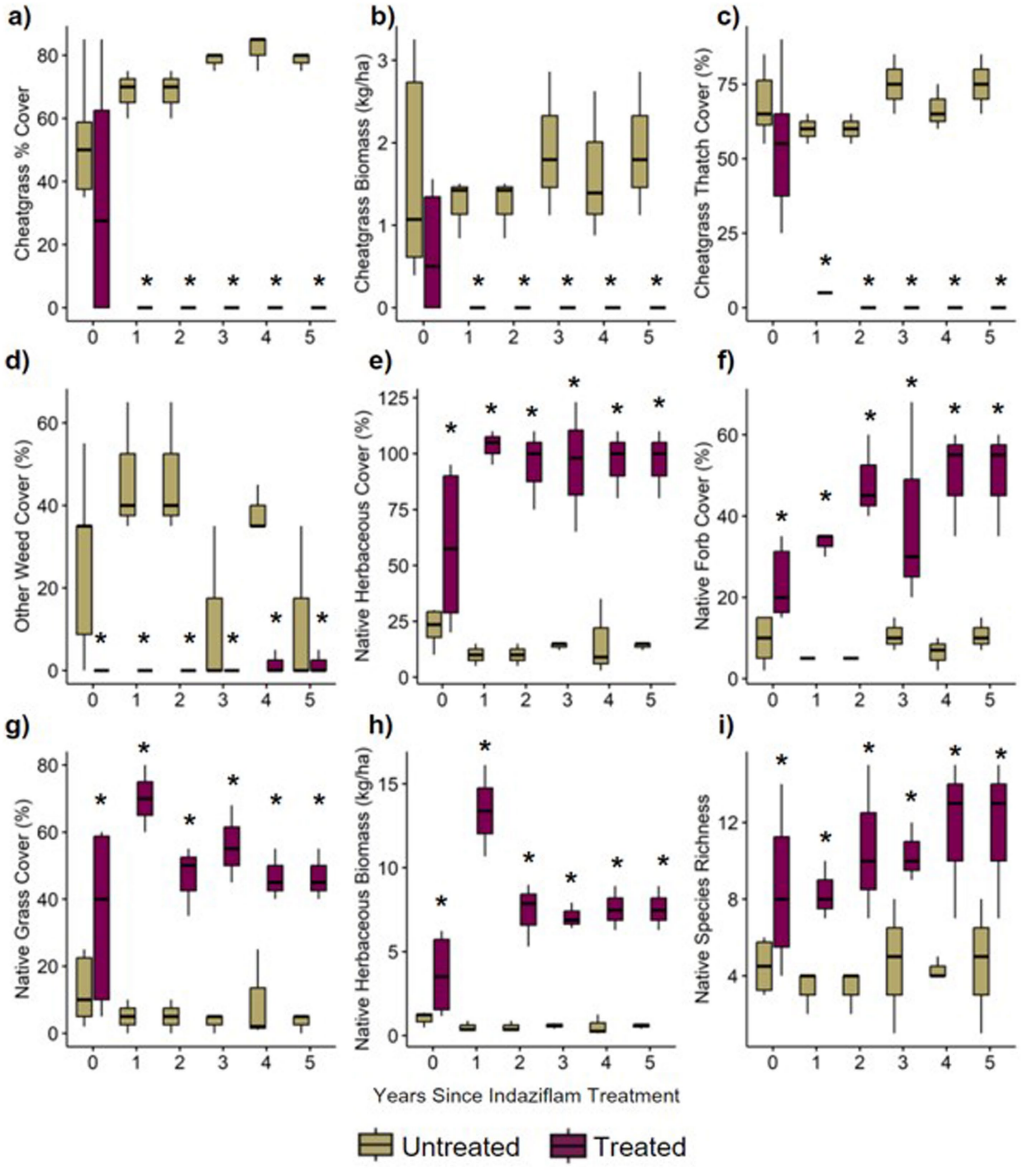
**(A)** percentage of the plot covered by cheatgrass, **(B)** biomass of cheatgrass collected in plot, **(C)** percentage of the plot covered by thatch (living and dead plant material) from cheatgrass, **(D)** percentage of the plot covered by weeds other than cheatgrass, **(E)** percentage of the plot covered by all native herbaceous material, **(F)** percentage of the plot covered by native forbs (subset of native herbaceous cover), **(G)** percentage of the plot covered by native grasses (subset of native herbaceous cover), **(H)** biomass of native biomass collected in plot, **(I)** count of native species in plot.

The results from generalized linear mixed models comparing herbicide treated vs. control plots showed striking differences in plant community composition (Figure 2). Overall, plots treated with indaziflam herbicide had substantially lower cheatgrass cover (Figures 2A–C) and higher native plant cover (Figures 2E–H) relative to untreated controls. These differences also varied in magnitude in paired plots observed 0–5 years following herbicide treatment (Figure 2). For cheatgrass cover, treated plots on average had ~75% lower cheatgrass percent cover, with an average of 0 % cheatgrass cover, relative to untreated controls (which at a significance level of *p* < 0.001 had a mean of 51–83% cover; Figure 2A). Cheatgrass thatch cover was also significantly lower in treatment plots overall (*p* < 0.001) and decreased slightly with increasing time since treatment (*p* = 0.086; Figure 2C). Total percent cover of other non-native weeds was also lower in treatment plots, reduced on average by 37% cover (*p* < 0.001; Figure 2D).

Treated plots on average had ~6-fold greater total native herbaceous plant cover relative to untreated controls (*p* < 0.001; treated average cover = >100%, control average cover = 20%; Figure 2E), with over 4-fold greater native forb cover (*p* < 0.001; Figure 2F) and 11-fold greater cover of native grasses in treated plots (*p* < 0.001; Figure 2G). Total herbaceous plant biomass (lb./ac) was higher (*p* < 0.001; Figure 2H) in treated plots. Native species richness was also substantially higher in indaziflam-treated plots (~10 versus ~4 native species per plot, respectively; *p* < 0.001; Figure 2I). Together, these results show steep declines in cheatgrass and weed cover in indaziflam-treated plots and increases in native plant cover and biomass in years following indaziflam application. All data from this project can be found at doi: 10.5061/dryad.7m0cfxq4r and in the NCBI database under the BioProject ID PRJNA1171444.

### Responses of soil physical characteristics to indaziflam treatment

**Figure 3:**
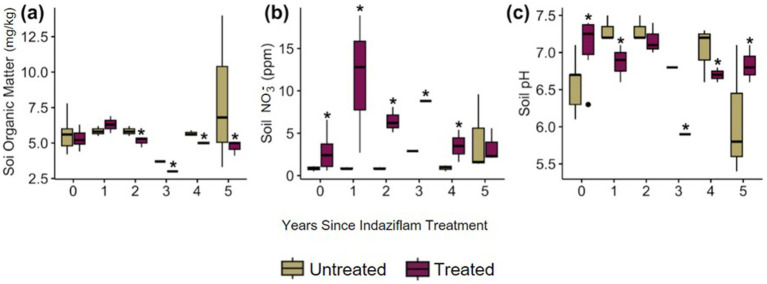
**(A)** concentration of soil organic matter (mg/kg) in soil samples from plot, **(B)** concentration of soil nitrate (ppm) in soil samples from the plot, **(C)** pH of the soil samples from plot.

Soil physical characteristics also differed between indaziflam treatment versus control plots. For soil properties, soil organic matter (SOM) was lower in indaziflam-treated plots (*p* = 0.033; Figure 3A) (*p* = 0.013; Figure 3A). While there were no significant differences in SOM in Year 0–1, in Years 2–5 SOM was significantly lower in treated plots. In contrast, soil nitrate (NO_3_^−^) was higher in indaziflam-treated plots (*p* < 0.001; Figure 3B). Although the magnitude of NO_3_^−^ ppm difference between treated and untreated plots was not consistent in time since treatment (*p* < 0.001; Figure 3B), soil NO_3_^−^ was significantly higher in treatment plots spanning all years since treatment except for in Year 5 (2022) where it was equivalent between treated and untreated plots. Finally, soil pH also differed across plots, although differences were variable across time since treatment (*p* = 0.010; Figure 3C). Soil pH was highly variable across years (Figure 3C). Soil physical characteristics and plant community data are indicated in the file “FINAL_BCPOSVeg.csv.”

### Microbial database results

16S data from this project resulted in 9,042 ASVs across all samples and treatments. Treated samples had 4,567 ASVs, and untreated samples had 4,589 ASVs in total (see file “FINAL16S_ASVs_ID.csv”). The most abundant phylum included the genus was *Nitrososphaeraceae* that represents ammonia-oxidizing archaea such as *Candidatus Nitrosocosmicus,* which are common in most soils (Sauder et al., 2017; Tourna et al., 2011). The second most abundant group were a part of the general *Bacillales* order, members of the *Firmicutes* phylum, which are also highly abundant and common soil microorganisms (Setlow and Doona, 2023).

ITS data resulted in 3,128 ASVs across all samples and treatments. Treated samples had 1,539 ASVs, and untreated samples had 1,450 ASVs in total (see file “FINALITS_ASVs_ID.csv”). The most abundant ASV was *Solicoccozymaeria*, a common soil fungus (Rui et al., 2022). *Mortierella*, another common soil fungus implicated in decay and organic matter cycling, was also abundant (Ozimek and Hanaka, 2021). Other information about community composition can be found in the Supplementary materials (file “Bradbury_BCPOS_SupplementaryMaterials.docx”).

### Microbial community composition variance

**Figure 4:**
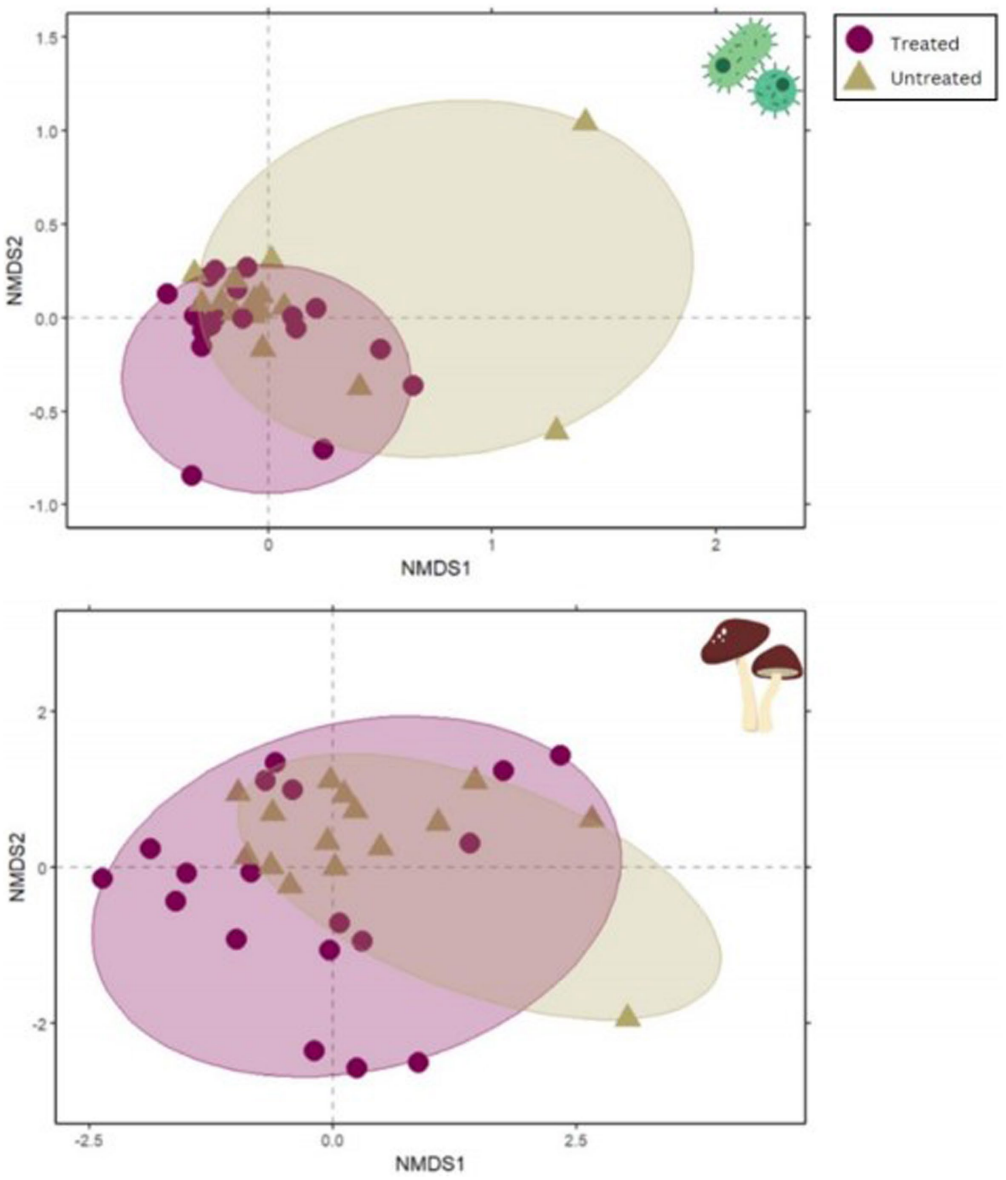
NMDS plots demonstrate the significant difference between treated and untreated sample microbial community composition. The plot on the top shows results from 16S and the lower plot shows ITS results. NMDS differences are visualized by distance between points. Circles represent 95% confidence intervals.

PERMANOVA testing nested by site demonstrated that plots treated with indaziflam significantly differed in microbial community composition from untreated plots for both bacteria/archaea (16S; *p* = 0.008; Table 2; Figure 4) and fungi (ITS; *p* = 0.001; Table 2; Figure 4).

**Table 2:** *p*-value and significance level of each soil and biomass variable in the MDRM test for 16S and ITS data showing that pH, organic matter, nitrate, and native forb biomass are significantly related to 16S and organic matter to ITS composition.

16S MDRM results			
Variable	Coefficient	*p*	Significance
pH	−0.024	0.040	**
OM	0.025	0.033	**
NO3-	0.006	0.084	*
Cheatgrass Biomass	−0.011	0.418	
Native Grass Biomass	0.008	0.062	
Native Forb Biomass	−0.014	0.08	*
Native Shrub Biomass	0.009	0.638	

ITS MDRM results			
Variable	Coefficient	*p*	Significance
pH	−0.002	0.708	
OM	0.008	0.068	*
NO3-	−0.002	0.202	
Cheatgrass Biomass	0.009	0.259	
Native Grass Biomass	0.004	0.151	
Native Forb Biomass	0.006	0.198	
Native Shrub Biomass	0.009	0.343	

Significance amount marked by symbols. *p < 0.05. **p < 0.005, ***p< 0.001.

Differences in Shannon diversity of bacterial/archaeal communities were also tested with ANOVAs and did not show a significant difference between treated and untreated sites in both 16S and ITS (Supplementary Table S1, *p* = 0.845). However, 16S had a much higher diversity overall than ITS.

### Multiple regression on distance matrices (MDRM)

The MDRM test indicated that soil pH (*p* = 0.040), OM (*p* = 0.033), NO_3_^−^ (*p* = 0.084), and native forb biomass (*p* = 0.080) were all significantly related to the difference in bacterial and archaeal community composition between treated and untreated plots. Only soil OM (*p* = 0.068) was marginally significantly related to the difference in fungal community composition (Table 2).

### Indicator species analysis results

Analysis of indicator species demonstrated that there were many ASVs significantly associated with indaziflam-treated and untreated plots. For 16S, 26 ASVs were indicative of treated sites, while 24 ASVs were indicative of untreated sites (see file “IndicSpecResults16S.csv”). ITS results had 4 ASVs significantly associated with treated sites and 24 ASVs associated with untreated sites (see file “IndicSpecResultsITS.csv”).

## Discussion

Cheatgrass invasion in dryland ecosystems across the western U.S. presents many challenges and considerations for land stewards. Indaziflam (Rejuvra™) has recently been approved for use to control cheatgrass. Yet, to date, there have been few studies of the herbicide’s effects on non-target organisms. Through plant surveys, soil physical analysis, and soil microbiome 16S/ITS amplicon sequencing, we found that treatment of cheatgrass with indaziflam had varied effects on different ecosystem actors in an arid foothill shrubland in Boulder County, USA.

This study found that plant community composition, soil physical characteristics, and soil microbiome shifted dramatically after indaziflam application. Cheatgrass cover was reduced by up to 80% in plots that had been sprayed just 1 year before monitoring, and native plants almost fully recovered in these plots. Organic matter was lower in treated plots than untreated plots, and soil nitrate (NO_3_^−^) was higher in treated plots than untreated plots, while pH was variable between treatments. Soil microbial communities were also impacted by indaziflam treatment, with bacterial and fungal communities being significantly different in composition between treated and untreated sites. Statistical analysis in this study also demonstrated a relationship between microbiome composition and soil organic matter, NO_3_^−^, pH, and native forb biomass.

These results show that indaziflam changes plant and soil dynamics in ecosystems, but more work is necessary to fully understand the mechanisms behind these shifts. This study showed that soil microbial communities were different between treated and untreated sites but did not parse out if the soil microbial communities were responding to the application or if they responded to plant community shifts. Based on the PERMANOVA statistical analysis that demonstrated soil microbiome is linked with soil physical characteristics and treatment, it is likely that both are true. However, understanding the mechanism of the soil microbiome shift, and the wider impacts of indaziflam through continuous monitoring, is critical for future research.

### Plant community composition and nutrient cycling shift in response to indaziflam treatment

Above ground, we found that application of indaziflam on cheatgrass-dominated plots successfully reduced cheatgrass cover by 80% on average. Native grasses and forbs recruited in plots that had been treated as recently 1 year prior, and native herbaceous plants covered up to 100% of treated plots. This response aligns with previous literature that has found similar dramatic decreases in cheatgrass and increases in native plant cover after indaziflam application (Alba et al., 2024; Courkamp et al., 2022; Arathi and Hardin, 2021; Kainrath et al., 2022; Meyer-Morey et al., 2021; Sebastian et al., 2017; Seedorf et al., 2022). Kainrath et al. (2022) showed that direct reduction of cheatgrass, as opposed to modifying soil physical and microbial characteristics, was the most effective restoration strategy for invaded areas and that native plants quickly recruited after removal in sagebrush habitat (Kainrath et al., 2022). Therefore, the dramatic reduction of cheatgrass after indaziflam application suggests that this tool could be useful for opening space for restoration to occur. Arathi and Hardin (2021) also found that native and pollinator-friendly plant species richness doubled a year after indaziflam application in another study conducted in Boulder, CO (Arathi and Hardin, 2021). This study, taken with the results of our study, demonstrates that the goal of native plant recruitment after cheatgrass invasion may be met with indaziflam application.

Belowground, we found that soil NO_3_^−^ increased after application and that SOM significantly decreased. Although Weber et al. (2015) also found that nitrate was significantly higher in native perennial rangeland than in cheatgrass-dominated areas, many prior studies have found more NO_3_^−^ after invasion, due to cheatgrass root exudation and leaf senescence (e.g., Baughman et al., 2016; Weber et al., 2015). Therefore, our more novel result that nitrate increased in the soil after indaziflam application could be due to either legacy effects of cheatgrass invasion or of increased nitrogen-synthesizing fungi that native plants partner within the rhizosphere. If nitrogen-synthesizing fungi are driving the increase in NO_3_^−^ after indaziflam application, this could point toward the restoration of relationships between native plants and microbes after cheatgrass removal.

The findings surrounding soil carbon have also been contradictory, and a recent review by Maxwell and Germino (2022) found that the carbon dynamics of sites across the Great Basin that have been invaded by cheatgrass vary from site to site and have high heterogeneity in the magnitude and direction of carbon change (Maxwell and Germino, 2022). Our study adds to the complex story of soil nutrient dynamics as cheatgrass invades and is removed, and native plants revegetate. Our result that soil organic matter decreased when cheatgrass was removed (Figure 3A) is likely because cheatgrass cover and thatch decreased so dramatically, and therefore, soil organic matter that had been previously added by the cheatgrass thatch, growth, and decomposition cycle was no longer present. Previous studies have shown that when plant cover decreases, soil organic carbon decreases, and as soil organic carbon comprises a large portion of SOM, our result that a decrease in cheatgrass cover may have led to a decrease in SOM agrees with this (Fekete et al., 2012; Koçak et al., 2021; Wan et al., 2019). These soil physical property and plant changes were also related to soil microbial community composition and shifts.

### Soil microbial community composition, but not diversity, differed significantly between treated and untreated sites

The results from soil community composition analyses (i.e., PERMANOVA and ANOVA Shannon diversity) demonstrate that indaziflam treatment significantly changed microbial community composition but not overall diversity. PERMANOVA testing showed a statistically significant difference in community composition between treated and untreated plots, indicating that microbes in the community are different in abundance and composition. Previous studies have shown that cheatgrass encroachment itself can shift soil microbial communities when compared to native states and that N_2_ fixing and uratolytic bacteria increasing while denitrifiers decrease is particularly indicative of cheatgrass dominance (Reitstetter et al., 2022). The indicator species analysis in this study mirrored this shift, meaning that cheatgrass removal was likely a major driver of nitrogen microbial dynamics. Furthermore, chiral herbicides such as indaziflam have been shown to move soil microbiomes toward aromatic compound digestion and affect nitrogen dynamics, which the results of our indicator species analysis also support (Asad et al., 2017; Pertile et al., 2021).

The results of the MDRM analysis provide insight into the ecological dynamics surrounding the difference in treated and untreated bacterial communities. Differences in bacterial and archaeal community composition were correlated with differences in soil organic matter, soil nitrate, soil pH, and native forb biomass, suggesting that associated ecosystem changes with indaziflam application are drivers of microbiome shift. This is a critical finding as it means that indaziflam application shifts ecosystem relationships, especially soil physical characteristics, which may have a cascading impact throughout the system. In contrast to bacterial communities, differences in soil fungal communities were only due to soil organic matter. The fact that soil fungal community composition was not significantly correlated with nitrate amount may mean that the observed nitrogen dynamics were related to native forb presence or that bacterial communities were doing the majority of fixation in the system. The decrease in organic matter that is potentially due to thatch cover reduction could be a key indicator of microbial community shift for both bacteria and archaea as well as fungi. Considering thatch cover and compensation for organic matter may be important for overall ecological restoration and management practices as this decrease having ripple effects on the microbial community could hinder restoration efforts. Furthermore, bacteria could be more sensitive to these shifts because of the number of variables that are associated with bacterial community change. Therefore, indaziflam’s effect on each of these variables may be critical for land managers to consider for ecosystem health post-application.

### Microbial indicator species differ between treated and untreated sites

Indicator species analysis demonstrated that the nitrogen metabolism, soil microbial characteristics, and community composition shifted after indaziflam application. Although the PERMANOVA and MDRM tests demonstrated the differences in microbial communities, the indicator species analysis showed key organisms that are significantly different between indaziflam-treated and untreated sites. In our indicator species analysis, ammonia-oxidizing bacteria, Nitrosomonadaceae, were found to be associated with untreated sites, while ammonia-oxidizing archaea, *Nitrososphaeraceae*, were found to be associated with indaziflam treatment. This could point to the soil microbiome becoming more similar to native community composition after cheatgrass is removed as Chen et al. (2021) found that ammonia-oxidizing bacteria were associated with disturbance and urbanization in the Southwestern U.S., while ammonia-oxidizing archaea were associated with undisturbed ecosystems. Other nitrogen dynamics of indaziflam application in the soil microbiome were indicated by the association of *Opitutaceae* and the fungi *Articulospora proliferata*, which engage in nitrate digestion and assimilation, in untreated sites, and of *Chloroflexi TK10* in treated sites, which digests N_2_ to nitrate (Chin et al., 2001; Johnston et al., 2019; Pöritz et al., 2015). *Microvirga* which are an unconventional root-associated nitrogen-fixing root-associated bacteria (Maheshwari and Sankar, 2022) were associated with treatment. Cheatgrass utilizes high amounts of nitrogen but does not make associations with nitrogen-fixing bacteria on their roots (Reitstetter et al., 2022). Therefore, *Microvirga*’s presence indicates that native plants that have grown after cheatgrass removal by indaziflam are likely associating with microbes, especially bacteria, to fix nitrogen in the soil (Erlacher et al., 2015). These differences in nitrogen metabolism of key taxa in the soil microbiome may give clues about nitrate dynamics of cheatgrass invasion as the nitrogen-fixing bacteria could be the drivers of our different result from past literature.

Other indicator species also shed light on the microbial community differences between treated and untreated sites. In treated sites, many significant indicator species specialize in different forms of chemical digestion. *Sphingomonas*, for instance, has been used in detoxification efforts because of its ability to digest organometals and support plant growth, while *Acidobacteriales* have been associated with acidic mining-contaminated soils (Kishimoto and Tano, 1987; Leys et al., 2004). Indaziflam is an aromatic compound, meaning that these organisms could be metabolizing the chemical itself. Furthermore, indaziflam is a fluoroalkyl triazine-containing compound. Previous study has tied *Acidobacteriales* to fluoroalkyl chemicals, and this order of bacteria’s presence could support the notion that some toxicity to the soil microbiome is occurring with cheatgrass application (Wang et al., 2023).

Finally, some ASVs associated with untreated and cheatgrass-dominated sites are indicative of ecological pH stress. Two of the bacterial indicator species for untreated sites, *namely, Blastocatellia* and *Acidimicrobiia*, both from the phylum *Acidobacteriota*, have been found to thrive in acidic soil (Tables 3, 4; Hazarika and Thakur, 2020; Huber and Overmann, 2018). Our results from soil testing showed that untreated sites had more acidic soil than treated sites on average, and the indicator species analysis showed that microbes may be responding to this pH shift. A connection between soil pH and indaziflam treatment is also supported by the MDRM analysis, which showed pH as significantly associated with bacterial and archaeal community composition. Ecologically, acidic soil can often be a cause for concern if it inhibits symbiosis between microorganisms and native plants or it changes plant recruitment (Zama et al., 2022), but since our result showed that native forbs grew well after treatment and made association with nitrogen-fixing bacteria, this may not be the case for our specific study system.

**Table 3:** Indicator species analysis of key 16S ASV differences between treated and untreated plots.

16S indicator species analysis results							
Phylum	Class	Order	Family	Genus	*p*	Significance	Ecological Function
Acidobacteriota	Acidobacteriae	Acidobacteriales	NA	NA	0.010	**	Found in acidic mine drainage, chemoorganotroph (Kishimoto and Tano, 1987)
Actinobacteriota	Thermoleophilia	Solirubrobacterales	67–14	NA	0.049	*	Decomposition of organic matter (Foesel et al., 2016)
Chloroflexi	TK10	NA	NA	NA	0.006	**	Nitrogen digestion (Speirs et al., 2019)
Crenarchaeota	Nitrososphaeria	Nitrososphaerales	Nitrososphaeraceae	NA	0.007	**	Archea, ammonia oxidation (Chen et al., 2021)
Proteobacteria	Alphaproteobacteria	Rhizobiales	Beijerinckiaceae	Microvirga	0.006	**	Lichen symbiosis, nitrogen fixation (Erlacher et al., 2015)
Proteobacteria	Alphaproteobacteria	Sphingomonadales	Sphingomonadaceae	Sphingomonas	0.020	*	Polycyclic aromatic hydrocarbon digestion (Leys et al., 2004)
Acidobacteriota	Blastocatellia	45,254	NA	NA	0.019	*	Characteristic of acidic soil (Huber and Overmann, 2018)
Actinobacteriota	Acidimicrobiia	NA	NA	NA	0.009	**	Indicative of acidic soil (Hazarika and Thakur, 2020)
Bacteroidota	Bacteroidia	Chitinophagales	Chitinophagaceae	NA	0.025	*	Ureolysis production, chitin digestion (Hasan, 2000, Wieczorek et al., 2019)
Proteobacteria	Gammaproteobacteria	Burkholderiales	SC-I-84	NA	0.001	***	Plant growth promotion (Yurgel et al., 2022)
Proteobacteria	Gammaproteobacteria	Burkholderiales	Nitrosomonadaceae	Ellin6067	0.031	*	Bacteria, ammonia oxidation (Chen et al., 2021)
Verrucomicrobiota	Verrucomicrobiae	Opitutales	Opitutaceae	Opitutus	0.033	*	Conversion of nitrate to nitrite (Chin et al., 2001)

Significance amount marked by symbols, *p < 0.05, **p < 0.005, ***p < 0.001.

**Table 4:** Indicator species analysis of key ITS ASV differences between treated and untreated plots.

ITS indicator species analysis results								
Phylum	Class	Order	Family	Genus	Species	*p*	Significance	Function
Basidiomycota	Agaricomycetes	Agaricales	Entolomataceae	Entoloma	byssisedum	0.050	*	Associated with wood decay, (He et al., 2019)
Ascomycota	Lecanoromycetes	NA	NA	NA	NA	0.009	**	Lichen indicative (Erlacher et al., 2015)
Basidiomycota	Agaricomycetes	Cantharellales	Ceratobasidiaceae	Rhizoctonia	NA	0.050	*	Plant pathogen, (Termorshuizen, 2007)
Ascomycota	Dothideomycetes	Pleosporales	Lentitheciaceae	Darksidea	NA	0.039	*	Associated with plant invasion in the desert southwest (Williams et al., 2022)
Ascomycota	Dothideomycetes	Pleosporales	Phaeosphaeriaceae	Phaeosphaeria	NA	0.005	**	Lignin degradation (Ferrari et al., 2021)
Ascomycota	Dothideomycetes	Pleosporales	Pleosporaceae	Pyrenophora	Sieglingiae	0.001	***	Plant pathogen (Masi et al., 2022)
Ascomycota	Leotiomycetes	Helotiales	Helotiaceae	Articulospora	Proliferata	0.001	***	Nitrogen assimilation decomposition (Johnston et al., 2019)
Ascomycota	Orbiliomycetes	Orbiliales	Orbiliaceae	Orbilia	NA	0.013	*	Nematophagy, (Kavitha et al., 2020)

Significance amount marked by symbols, *p < 0.05, **p < 0.005, ***p < 0.001.

Fungi indicator species suggest improvements to soil health following indaziflam application. Untreated sites were associated with several fungi that are plant pathogens and associated with plant invasion in the desert southwest, such as *Darksidea* and *Pyrenophora*, while treated sites were associated with lichen-indicative fungus (Erlacher et al., 2015; Masi et al., 2022; Termorshuizen, 2007; Williams et al., 2022). The fact that plant pathogens were associated with untreated sites may mean that indaziflam treatment lowers plant pathogen presence, although the mechanism by which this occurs is unclear. The indication of lichen-associated fungi after treatment may be particularly important as lichen was visually observed to be growing on the soil more frequently in treated sites. Lichens have been shown to be important for soil stability, moisture, and overall health, and therefore, this association may demonstrate an increase in soil condition after treatment (Finger-Higgens et al., 2022).

### Study limitations and future research opportunities

Although this study is among the first to evaluate critical non-target impacts of indaziflam, it has several limitations. First, this study sampled sites within a relatively small spatial extent, with limited replicates and spatial homogeneity. More repetition, ecosystem representation, and in-depth sampling are needed in the future. Furthermore, this study was retrospective in its temporal aspect, and a continuous monitoring effort is needed for changes over time to be characterized. We suggest that future studies investigate impacts on other non-target organisms, nitrogen mechanics after application, and how the soil microbiome may be processing indaziflam.

### Indaziflam application shifts soil microbiome, physical characteristics, and plant community composition: implications for dryland management and restoration

As cheatgrass dominance in the Intermountain West poses increasing risks to human and ecological communities, informed management decisions about restoration of cheatgrass-dominated communities have become increasingly important. This study found that indaziflam did successfully remove cheatgrass from an arid foothill shrubland ecosystem and that native plant communities grew back after this removal. However, this study also found that decreases in organic matter and increases in soil nitrate may be related to indaziflam treatment. These soil physical property changes have relationships with the soil microbiome. The results suggest that the soil microbiome was significantly altered by indaziflam application and that these changes may be related to changes in soil organic matter, nitrate, pH, and native forb biomass associated with indaziflam treatment, meaning that land managers may consider assessing soil biogeochemistry before application. Finally, indicator species analyses showed that nitrogen dynamics may particularly change after indaziflam application. The shift in microbial community may be beneficial, as indicated by the presence of lichen-associated and pH-neutral microbes in treated sites, but further research is needed to assess long-term impacts of the observed microbial shift. These results are critical for land managers trying to restore and steward lands that have been invaded with cheatgrass, as assessing whether to use indaziflam to reduce cheatgrass cover may have nuanced effects on the soil biogeochemistry and microbial ecology of the ecosystem.

## Data Availability

The datasets presented in this study can be found in online repositories. The names of the repository/repositories and accession number(s) can be found: https://www.ncbi.nlm.nih.gov/bioproject/1171444, PRJNA1171444.

## Footnotes

^1^ http://plants.usda.gov

^2^ https://agsci.colostate.edu/soiltestinglab/

^3^ https://github.com/yhwu/idemp
