# Supplementary material for: Plant and soil microbial composition legacies following indaziflam herbicide treatment
Source: Front Microbiol. 2024 Dec 18;15:1450633. doi: 10.3389/fmicb.2024.1450633 (PMC11688475; doi:10.3389/fmicb.2024.1450633)
Supplement: Supplementary file 1 [file Data_Sheet_1.pdf]

SUPPLEMENTARY MATERIAL

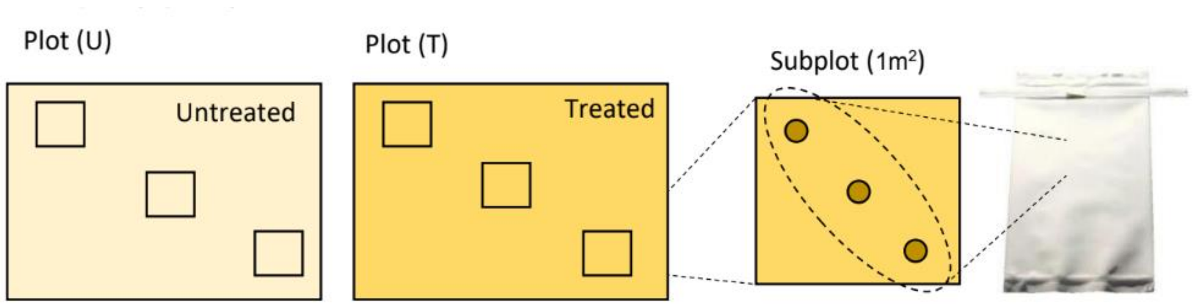

Figure S1. *Subplot design of soil sampling method.*

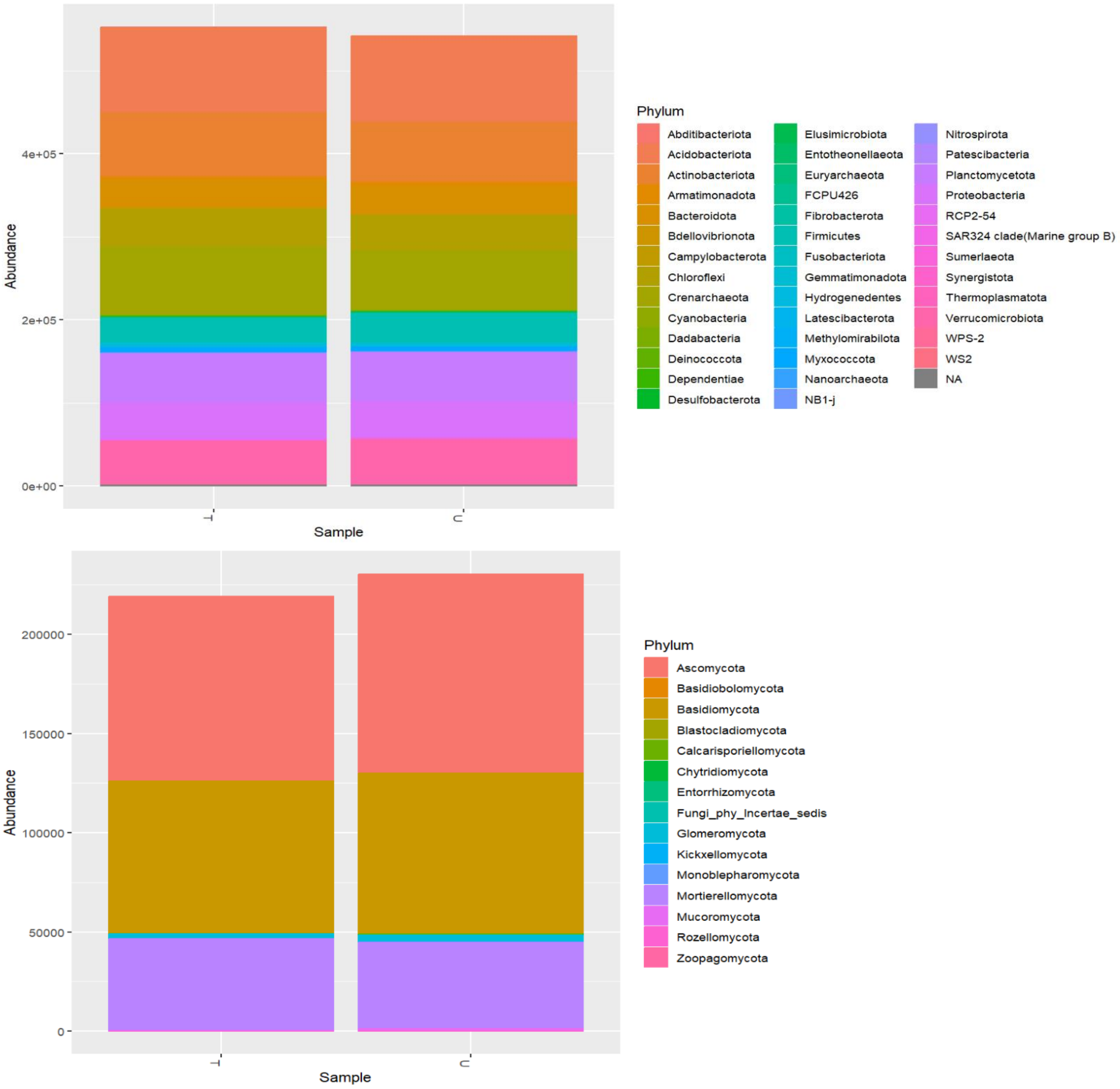

Figure S2. *Bacterial and fungal community composition between treated (T) and untreated (U) sites with abundance.*

	Untreated		Treated	
**Site ID**	**16S**	**ITS**	**16S**	**ITS**
DORO 2020	432.22	30.44	410.16	50.74
DORO 2021	432.22	59.06	364.02	39.63
RABB 2022	441.67	37.39	436.04	31.81
TREVA 2017	399.38	59.06	425.22	42.54
TREVA 2018	432.79	48.22	444.74	49.35
TREVA 2022	434.06	52.00	464.08	40.16

Table S1. *Averaged 16S and ITS Shannon index and for each site and treatment. Higher indices indicate more diversity.*

a)

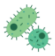

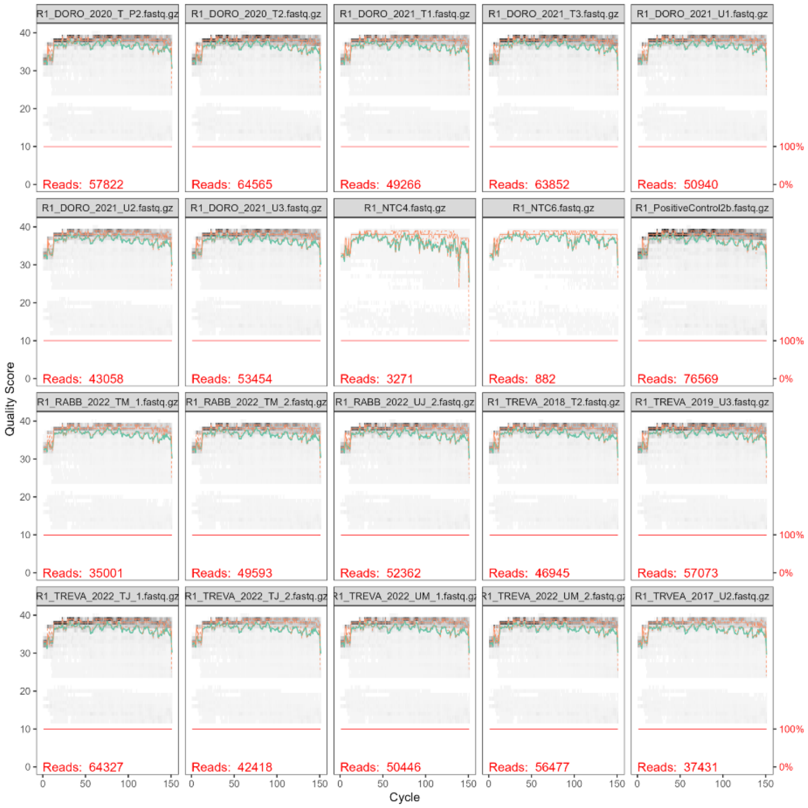
R1_DORO_2020_T_P2.fastq.gz
R1_DORO_2020_T2.fastq.gz
R1_DORO_2021_T1.fastq.gz
R1_DORO_2021_T3.fastq.gz
R1_DORO_2021_U1.fastq.gz
Reads: 57822
Reads: 64565
Reads: 49266
Reads: 63852
Reads: 50940
R1_DORO_2021_U2.fastq.gz
R1_DORO_2021_U3.fastq.gz
R1_NTC4.fastq.gz
R1_NTC6.fastq.gz
R1_PositiveControl2b.fastq.gz
Reads: 43058
Reads: 53454
Reads: 3271
Reads: 882
Reads: 76569
R1_RABB_2022_TM_1.fastq.gz
R1_RABB_2022_TM_2.fastq.gz
R1_RABB_2022_UJ_2.fastq.gz
R1_TREVA_2018_T2.fastq.gz
R1_TREVA_2019_U3.fastq.gz
Reads: 35001
Reads: 49593
Reads: 52362
Reads: 46945
Reads: 57073
R1_TREVA_2022_TJ_1.fastq.gz
R1_TREVA_2022_TJ_2.fastq.gz
R1_TREVA_2022_UM_1.fastq.gz
R1_TREVA_2022_UM_2.fastq.gz
R1_TRVEA_2017_U2.fastq.gz
Reads: 64327
Reads: 42418
Reads: 50446
Reads: 56477
Reads: 37431
Quality Score
Cycle
100%
0%

b)

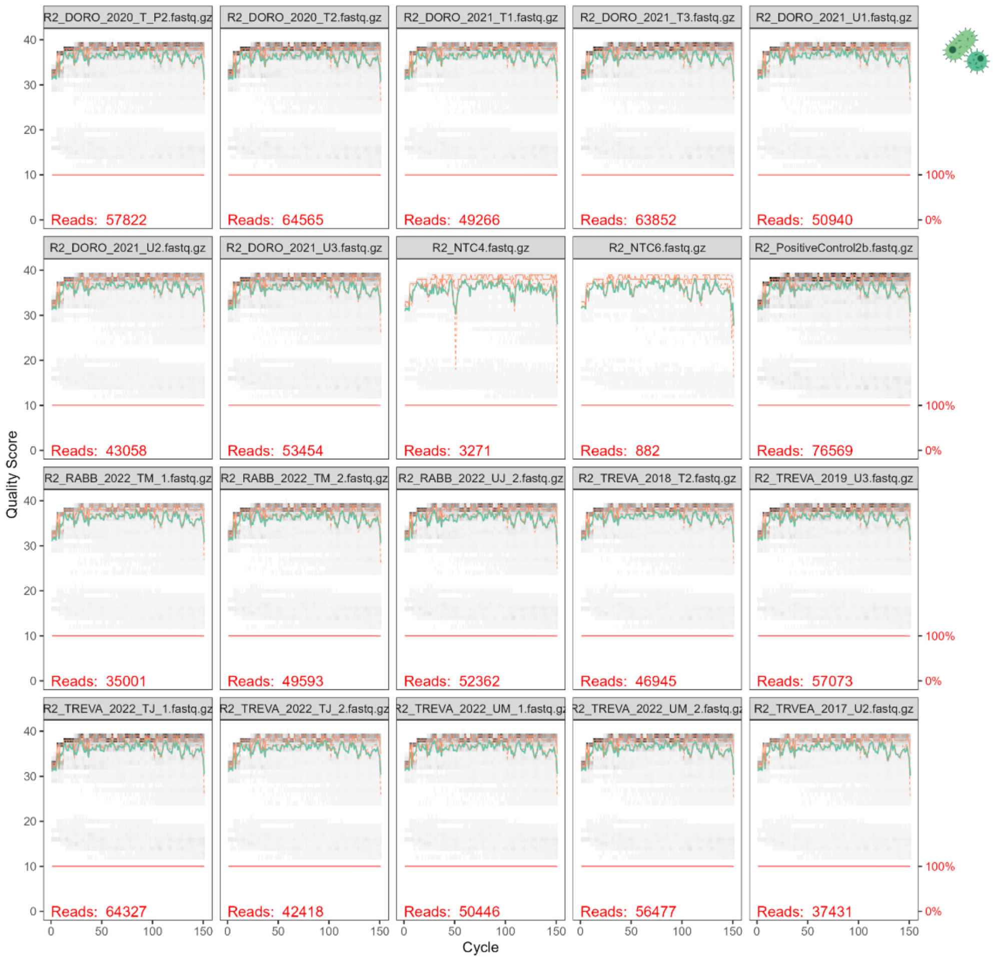

R2_DORO_2020_T_P2.fastq.gz
Reads: 57822
R2_DORO_2020_T2.fastq.gz
Reads: 64565
R2_DORO_2021_T1.fastq.gz
Reads: 49266
R2_DORO_2021_T3.fastq.gz
Reads: 63852
R2_DORO_2021_U1.fastq.gz
Reads: 50940
R2_DORO_2021_U2.fastq.gz
Reads: 43058
R2_DORO_2021_U3.fastq.gz
Reads: 53454
R2_NTC4.fastq.gz
Reads: 3271
R2_NTC6.fastq.gz
Reads: 882
R2_PositiveControl2b.fastq.gz
Reads: 76569
R2_RABB_2022_TM_1.fastq.gz
Reads: 35001
R2_RABB_2022_TM_2.fastq.gz
Reads: 49593
R2_RABB_2022_UJ_2.fastq.gz
Reads: 52362
R2_TREVA_2018_T2.fastq.gz
Reads: 46945
R2_TREVA_2019_U3.fastq.gz
Reads: 57073
R2_TREVA_2022_TJ_1.fastq.gz
Reads: 64327
R2_TREVA_2022_TJ_2.fastq.gz
Reads: 42418
R2_TREVA_2022_UM_1.fastq.gz
Reads: 50446
R2_TREVA_2022_UM_2.fastq.gz
Reads: 56477
R2_TRVEA_2017_U2.fastq.gz
Reads: 37431
Quality Score
Cycle
100%
0%

c)

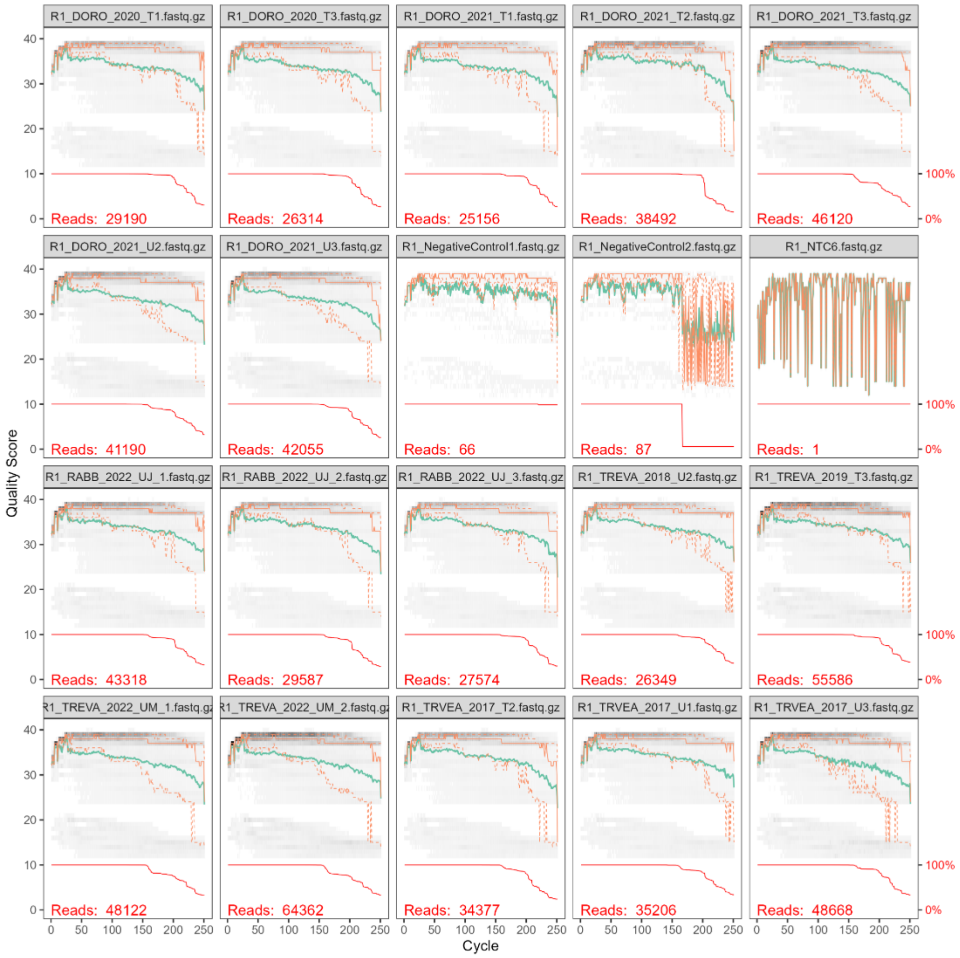
R1_DORO_2020_T1.fastq.gz
R1_DORO_2020_T3.fastq.gz
R1_DORO_2021_T1.fastq.gz
R1_DORO_2021_T2.fastq.gz
R1_DORO_2021_T3.fastq.gz
Reads: 29190
Reads: 26314
Reads: 25156
Reads: 38492
Reads: 46120
R1_DORO_2021_U2.fastq.gz
R1_DORO_2021_U3.fastq.gz
R1_NegativeControl1.fastq.gz
R1_NegativeControl2.fastq.gz
R1_NTC6.fastq.gz
Reads: 41190
Reads: 42055
Reads: 66
Reads: 87
Reads: 1
R1_RABB_2022_UJ_1.fastq.gz
R1_RABB_2022_UJ_2.fastq.gz
R1_RABB_2022_UJ_3.fastq.gz
R1_TREVA_2018_U2.fastq.gz
R1_TREVA_2019_T3.fastq.gz
Reads: 43318
Reads: 29587
Reads: 27574
Reads: 26349
Reads: 55586
R1_TREVA_2022_UM_1.fastq.gz
R1_TREVA_2022_UM_2.fastq.gz
R1_TRVEA_2017_T2.fastq.gz
R1_TRVEA_2017_U1.fastq.gz
R1_TRVEA_2017_U3.fastq.gz
Reads: 48122
Reads: 64362
Reads: 34377
Reads: 35206
Reads: 48668
Quality Score
Cycle
100%
0%

d)

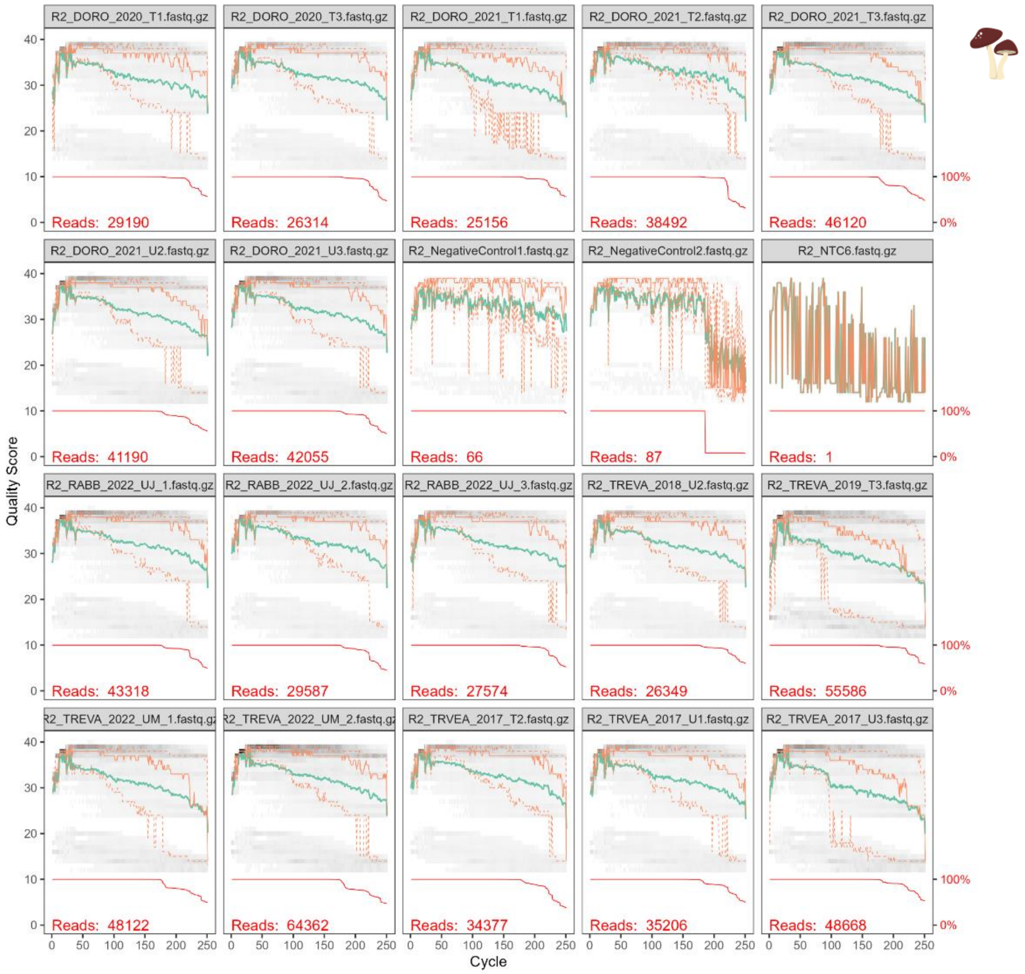

***Figure S3**. Quality read plots from sequencing. a) 16S forward reads, b) 16S reverse reads, c) ITS forward reads, d) ITS reverse reads.*

Vegetation and Soil Physical Characteristic Linear Model Results

Variable	Coefficient	p	Significance
Cheatgrass Cover	0.073	0.114	
Native Herbaceous Cover	-0.046	<0.001	***
pH	0.133	0.821	
SOM	0.027	0.623	
NO3	-0.526	0.007	*

Table S2. *Linear model results for vegetation shifts with treatment. Intercept estimate comparing treated to untreated and p-value (p) are included. Significance amount marked by symbols, * = < 0.1, ** = <0.05, *** = <0.005.*

PERMANOVA Results

Gene	R2	p	Significance
16S	0.0411	0.008	**
ITS	0.0465	0.001	***

Table S3. *PERMANOVA model results for differences in microbial composition, nested within each site. Correlation coefficient (R2) and p-value (p) are included. Significance amount marked by symbols, * = < 0.1, ** = <0.05, *** = <0.005.*

Appendix S1. Full description of microbial sequencing and statistical analysis methodology.

Soil DNA extraction, PCR, and gene amplicon sequencing

First, DNA was extracted from 0.25 grams of the 37 soil samples using the Qiagen DNeasy PowerSoil Pro Kit (Qiagen, Hilden, Germany). Next, we used 515F/806R primers to amplify the V4 region of the 16S rRNA gene for bacteria and archaea (Apprill et al., 2015; Caporaso et al., 2011; Parada et al., 2016), and ITS1-F/ITS2 primers for the ITS gene region for fungi (Bellemain et al., 2010; Smith & Peay, 2014). Each sample was assigned a 12-bp barcode, homogenized, and then randomly assigned a location on a 96-well plate. Four blank samples were included as negative controls. Two samples with known microbial composition were included as positive controls (from Kimmel, et. al, in review). Duplicated PCR reactions were run for all samples using Invitrogen's Platinum II Hot-Start PCR Master Mix (Invitrogen, Waltham, MA). After confirming amplification and length via gel electrophoresis, amplicons were then normalized using the ThermoFisher Scientific SequalPrep Normalization plates (Thermo Fisher Scientific Inc. USA). Both libraries were then sequenced with the Illumina MiSeq platform. The 16S library was sequenced using a 300-cycle v2 paired end kit and the ITS was sequenced using a 500-cycle v2 paired end kit. Both runs included a 15% phiX spike.After sequencing, reads were demultiplexed with idemp (idemp; https://github.com/yhwu/idemp) and adaptors were trimmed using cutadapt (Martin, 2011).

We then used the dada2 package in R (Callahan et al., 2016) to characterize the microbial communities in each sample. First, we used the filterAndTrim() function (settings: 16S truncLen = c(150,150), ITS truncLen = c(200,220), maxEE = (2,2), truncQ = 2, rm.phix = T) to trim all sequences to the same length by filtering based on the number of ambiguous bases, a minimum quality score, and the expected number of errors in the read. Next we learned error rates from 1x10^8 bp chosen from a random subset of the samples. Then we used the derepFastq() function

to depreplicate the sequences, which output a list of unique sequences and their abundances, where identical sequences were grouped together. We next applied a denoising algorithm using the dada() function. This involved partitioning the sequences where the most abundant sequence was made the center of the partition, and then all sequences were compared to the center. Sequences were first compared based on kmer distance and banded alignment. Then an error rate was calculated based on differences in bases between sequences, cross-referenced with quality scores. These error rates then allowed for the calculation of abundance p-values, where low values indicate that a certain sequence is too abundant to be considered an error in sequencing, and will then get partitioned out of the algorithm as a new taxonomic unit (here we used amplicon sequence variants, or ASVs). After the partitioning algorithm was run, we used the isBimeraDenovo() function to identify and remove bimeras (two-parent chimeric sequences), and the mergePairs() function to merge paired forward and reverse reads. Finally, we used a Bayesian taxonomic identifier as implemented in the dada2 package to assign a taxonomy based on UNITE (Oct. 2021 release for ITS) and Silva (v 138.1 for 16S).

Statistical Analyses

The microbiome data was filtered and rarified prior to statistical analysis. The 16S data was first filtered to exclude ASV's where the phylum was not able to be identified, as well as samples that had below a 4000 read count, and ASV's that were chloroplasts or mitochondria. Chloroplasts and mitochondria were removed because these organelles are only found in plants and animals, and their presence indicates contamination from a species outside of the microbial community. Further, ASV's that were highly abundant in negative control samples were filtered out after verifying that these ASV's were not highly abundant in the sample data. The data was

then rarified to 22,385 reads, which was the lowest read count in the 16S dataset. The same process (without chloroplast and mitochondria exclusion) was followed with the ITS dataset.

All statistical analyses were performed in R version 4.2.2 (R Core Team, 2023). To evaluate the effects of indaziflam treatment on native and exotic plant cover and biomass and soil physical characteristics relative to untreated control plots, we used generalized linear mixed effects models (GLMMs). We built these predictive GLMMs for all plant and soil response variables using the lme4 package in R (Bates et al., 2014). Separate modules were built for the following response variables: cheatgrass cover (%), cheatrass biomass (ounces per m2), cheatgrass thatch depth (cm), total native herbaceous plant cover (%), total native forb cover (%), total native shrub cover (%), native species richness, and soil mineral nutrient levels of interest (i.e., organic matter, NO_3^- (ppm), pH, others). In each model, indaziflam treatment (i.e., treated vs control) and time since treatment (in years; i.e., 0-5) were included as fixed effects and Site_ID was included as a random effect. The significance of individual terms ($p < 0.05$) included in final models was estimated using a Wald Type II X2 test ('ANOVA' function, car package; Fox et al., 2012). For significant variables, we used planned contrasts to explore differences in group means among levels using the 'emmeans' function (package emmeans; Lenth & Lenth, 2018).

To analyze differences in soil microbial community composition between treated and untreated plots, we used PERMANOVA (permutational analysis of variance) and PERMDISP (permutations of dispersion) tests using the using adonis2(), pairwise.adonis2(), betadisper(), and permutest() functions of the vegan, pairwiseAdonis, and smartsnp packages (Herrando-Pérez et al., 2021; Oksanen, 2020; Polanco-Martínez, 2020). In these models, treatment (treated with indaziflam or not treated) was the predictor variable, and Bray-Curtis dissimilarity matrices of vegetation and soil variables were the response variables for both bacteria/archaea and fungi

communities. PERMANOVAs were nested by site, as high variability between sites would skew results without nesting. To compare differences in diversity between treated and untreated plots within each site, we first calculated the Shannon index and species richness for each sample using the diversity() and specnumber() functions of the vegan package (Oksanen, 2020).

We then used ANOVA (analysis of variance) tests to compare the Shannon indices and species richness', first making sure the data met assumptions of normality and equality of variances using the shapiro.test() and leveneTest() functions of the car package (Fox, 2023). To parse apart if soil and ecological variables had a related effect on microbial diversity and community composition, we also conducted a multiple regression on distance matrices (MDRM) test (Goslee & Urban, 2007). To ensure that correlated variables were not included in the MDRM, the cor() function, which is in the corrplot package, was used to assess correlation between soil and plant variables (Wei & Simko, 2021). Variables correlated to a level of 0.6 or higher were not included together in the analysis. It was found that biomass plant variables (cheatgrass, native forb, native grass, and native shrub) were only weakly correlated, and therefore, these were chosen to represent plant community effect on the soil microbial community. Soil physical characteristics of soil organic matter, nitrate, and pH were chosen to be included in the MDRM because of a priori hypotheses about these variables and the fact that they were not correlated to each other. Finally, we performed an indicator species analysis to determine taxa indicative of treated and untreated conditions. For this analysis we used the multipatt() function of the indicspecies package (De Cáceres et al., 2010).
